# Improving the Efficiency of an Emergency Department Based on Activity-Relationship Diagram and Radio Frequency Identification Technology

**DOI:** 10.3390/ijerph16224478

**Published:** 2019-11-14

**Authors:** Shao-Jen Weng, Ming-Che Tsai, Yao-Te Tsai, Donald F. Gotcher, Chih-Hao Chen, Shih-Chia Liu, Yeong-Yuh Xu, Seung-Hwan Kim

**Affiliations:** 1 Department of Industrial Engineering and Enterprise Information, Tunghai University, Taichung 40704, Taiwan; sjweng@thu.edu.tw (S.-J.W.); p8311011@gmail.com (C.-H.C.); liushihchia@gmail.com (S.-C.L.); 2 Healthcare Systems Consortium, Tunghai University, Taichung 40704, Taiwan; 3 Institute of Medicine and School of Medicine, Chung Shan Medical University, Taichung 40201, Taiwan; 4 Emergency Department of Chung Shan medical university hospital, Taichung 40201, Taiwan; 5 Department of International Business, Feng Chia University, Taichung 40724, Taiwan; 6 Department of International Business, Tunghai University, Taichung 40704, Taiwan; dongotcher@yahoo.com; 7 Department of Computer Science and Information Engineering, Hungkuang University, Taichung 43302, Taiwan; yyxu@sunrise.hk.edu.tw; 8 Department of Business Administration, Ajou University, Suwon 443-749, Korea; seunk@ajou.ac.kr

**Keywords:** hospital operations, radio frequency identification, facility layout design, activity-relationship diagram, simulation

## Abstract

Emergency department crowding has been one of the main issues in the health system in Taiwan. Previous studies have usually targeted the process improvement of patient treatment flow due to the difficulty of collecting Emergency Department (ED) staff data. In this study, we have proposed a hybrid model with Discrete Event Simulation, radio frequency identification applications, and activity-relationship diagrams to simulate the nurse movement flows and identify the relationship between different treatment sections. We used the results to formulate four facility layouts. Through comparing four scenarios, the simulation results indicated that 2.2 km of traveling distance or 140 min of traveling time reduction per nurse could be achieved from the best scenario.

## 1. Introduction

Overcrowding in Emergency Departments (ED) of hospitals has been examined extensively [1]. EDs in hospitals need to put in place efficient systems that minimize costs and also yield satisfactory levels of care. This investigation attempted to develop and deploy Discrete Event Simulation (DES) to monitor nurse “traffic” patterns to identify potential bottlenecks, ameliorate inefficiencies, optimize flows, and identify solutions for reducing patient time in the ED while also seeking to maximize patient satisfaction. Hospital managers can make use of the scheme data as a realistic representation to examine different inputs and outputs and make alterations which best fit hospital operations. The proposed techniques used here such as an activity-relationship diagram and radio frequency identification applications (RFID) have been applied in the manufacturing industry widely, however, none of the previous research adopted RFID technology along with the concept of facility redesign to improve medical operation flows in the healthcare industry. Therefore, we would like to test the proposed techniques in a hospital. In this case, we collaborated with a hospital in Taiwan. Finally, we would like to focus on the improvement of ED operations.

In this study, we proposed reallocating ED resources and modifying its facility layout. We intended to approach this research in three ways: First, we used discrete-event simulation (DES) to identify the structure of ED operations and ED patient treatment flow. After simulations were performed and analyzed, facility resources could be modified based on differential scenarios. Second, the data for the DES would be derived from the collection and analysis of nurse traffic patterns through ED via RFID. Third, we would use the results of the simulation to propose modifications of the design of the ED facilities in an effort to emphasize optimal efficiency, to smooth motion flow, and to maximize patient care.

## 2. Literature Review

ED crowding, a consequence of simultaneously increasing demand for health care and a deficit in available hospital beds and ED beds, has become an increasingly significant public health problem [2,3,4,5]. ED crowding has been described as both a patient safety issue and a worldwide public health concern [6,7]. ED overcrowding negative effects include deleterious patient outcomes (such as long wait times, reduced quality of care treatment delays, and increased numbers of patients who leave without being seen), as well as negative financial consequences [8,9,10]. Crowding is a problem in the United States where one study cited its prevalence as 12–73% of the time [11] and was also an international problem [12]. Similarity, significant overcrowding is extant in Taiwan [13].

As in many other parts of the world, overcrowding in Taiwan hospital EDs is an increasingly scrutinized area. ED admissions in Taiwan increased by 11.8% from 2008 to 2018 [14]. This rise in the total emergency room population and the resulting longer wait times have resulted in overcrowding.

### 2.1. Simulation

EDs in Taiwan hospitals must implement efficient systems that minimize costs while also providing satisfactory levels of care. As noted in the Introduction above, we proposed reallocating ED resources and modifying the layout based on nurse movement pattern simulations. Recent articles in the literature have focused on reducing waiting durations and hiking service performance (throughput) by upgrading the actual framework [15]. Of late, the use of simulation-based studies has been extended to answer “what if” questions, endeavoring to design more efficacious healthcare services [16]. DES is a method that avoids the use of states and fixed cycle lengths and instead models events at the individual level [17]. Simulation is utilized to examine critical parts of healthcare processes such as facility design (EDs, ORs, etc.), staff arrangement and scheduling, and bed capacity operations [18,19]. Using simulation, [20] conducted a study of an ED in a British hospital, with the objective of determining the impact of key resources (waiting times, waiting lines, and throughput). Another recent study developed an original DES model to examine crowding and patient flow for staffing decision-making at an urban academic ED and serves as a practical example in this area [19].

Simulation (specifically DES) is applicable in a healthcare venue for this reason: Crowding is a complicated phenomenon that can be encapsulated through a variety of metrics including the volume of waiting patients, boarding patients, or occupied beds. Other forecasting approaches necessitate the selection of a dependent variable prior to model development; conversely, DES can yield a detailed list of patients forecast to be in the ED subsequently, and from this data, the outcome measures can be derived [21].

Thus, DES indeed collects data and analyzes output, while correspondingly understanding the complexity of the system and plotting a valid model to be used in maximizing managerial decisions [22]. The strength of DES is the ability to include system details, time dependent actions, and system drawbacks. DES allows decision makers to draw on data about system results as well as insights into the effects of altered conditions across time [23].

### 2.2. Nurse Movement Analysis Through the Emergency Department

In one recent example of a simulation study [24], a computer-based simulation tool was used to investigate the impact of decentralization of nursing support spaces on nurses’ walking distances and their unproductive use of time. Several studies [25,26] have noted nurses taking many paths during a work shift and little is known about the number, purpose, or meaning of the many workflow paths a nurse takes to perform their role. Furthermore, the literature has suggested that more than a quarter of a nurse’s time in a nursing home was spent walking [27,28,29]. Research points to less walking time contributing to more patient care activities and better patient outcomes [27,30]. This being the case, we proposed observing nurse traffic patterns using simulation in an effort to analyze the effects of changing their movements throughout the layout of the emergency department.

Today, the RFID system is being used successfully in the areas of manufacturing, supply chain, agriculture, transportation, healthcare, and services [31,32]. RFID applications have been used in data acquisition [33,34,35], queuing analysis of hospital systems [36,37], and tracking patients [33,38]. The growing sophistication of computers and software should allow information technology to play a vital part in streamlining care, catching and correcting errors, assisting with decisions, and providing feedback on performance [32]. Promising benefits related to the implementation of RFID in healthcare were patient safety, patient and asset tracking, efficiencies in patient care, and provider satisfaction [39]. Consequently, it is appropriate and advantageous to RFID to acquire the data to input into our simulation models with the ultimate goal of modifying the design of the ED facilities in an effort to emphasize optimal efficiency, to smooth motion flow, and to maximize patient care. As was noted in [40], using these measurements, managers and supervisors can help plan nurses’ shifts more efficiently, and designers and architects can use the information to design more efficient hospital units.

## 3. Methodology

We proposed a hybrid approach to reallocate emergency resources and redesign facility layout. There were three stages to our approach. The first stage was to use a DES model for developing the base framework of ED operations and ED patient treatment flow. In DES, we are able to capture the complexity and uncertainty of parameters of an ED system such as the proportion of each triage and acuity scale and the different processes of patient flows. Through the DES scenario analysis, resources would be reallocated strategically.

The second stage was to collect and analyze nurses’ movement patterns through RFID applications. The RFID applications are used to facilitate the investigation of the association between motion study and bedside care time and the impact of bedside care time on the prognosis within the work environment. One of the advantages of the RFID applications is to trace and identify multiple nurses’ travel movements and time effectively with minimum human resource usage [41]. In this stage, the transit time, distance traveled, and travel route over time between each emergency station was collected and analyzed in our DES model.

**Figure 1:**
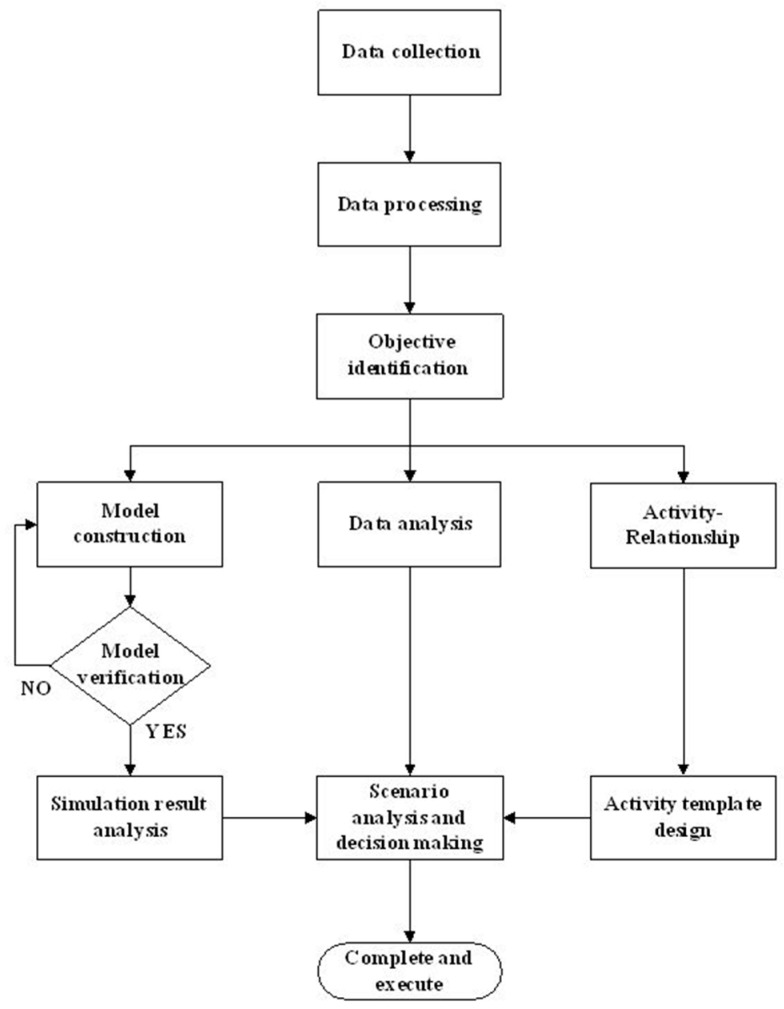
Research Framework.

The third stage involved the redesigning of the facilities. We first determined the correlation between each emergency facility according to the discussions with the emergency staff. Next, we used the activity-relationship diagram to diagnose the facility layout. The results of each modification of the layout were simulated in our DES model for retrieving the optimized ED resource allocation and facility layout. The proposed research framework is shown in Figure 1.

### 3.1. Emergency Department Patient Flows

**Figure 2:**
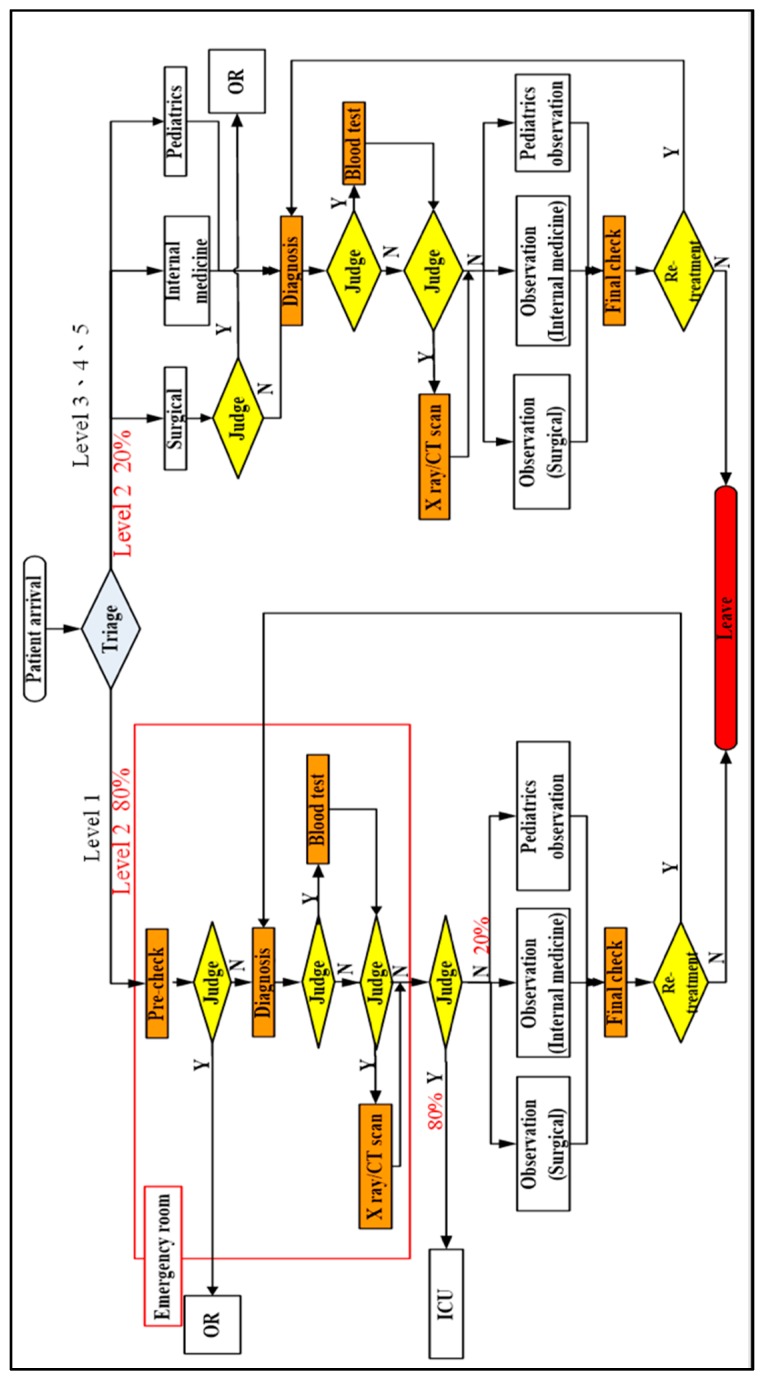
Emergency Department patient treatment flow.

In this section, we discuss the patient flow in the ED of Chung Shan Medical University Hospital. After the patients arrived in the ED, they would be classified and diagnosed using the Five-Level Taiwan Triage and Acuity Scale. The scale based on the severity of the patient injury is for identifying the priority of the treating sequence. There were five acuity levels in descending order: level 1 (resuscitation), level 2 (emergent), level 3 (urgent), level 4 (less urgent), and level 5 (not urgent). The first step of diagnosis was that a level 1 patient and 80% of the level 2 patients were sent to the emergency room and they would be checked if a surgery was needed. 20% of the level 2 patients and the rest of the patients would go to the surgical, internal medicine, or pediatrics consulting room. The second step was that patients would be checked, given blood and urine tests, and/or further checked by magnetic resonance imaging (MRI), *X*-ray or other tests. Next, the doctor judged if the patients were to be kept under observation or were admitted. These patients would then be sent to the first observation section (internal medicine), second observation section (internal medicine), surgical observation section, pediatrics observation section, or temporary observation section until they were admitted or left. The ED patient flow diagram is shown in Figure 2.

### 3.2. Simulation Data Collection and Model Setting

In the simulation model, historical ED patient data were provided by Chung Shan Medical University Hospital. We generated some inputs of the parameters with no records based on nurses’ suggestions. The initial model of 365 days with a 24-h period was developed in Simul8. There were 47,163 records in the dataset. The model inputs and assumptions are summarized in Table 1 and Table 2. For privacy protection, the study was approved by the Institutional Review Board (IRB) of Chung Shan Medical University Hospital (CSMUH No: CS17012).

### 3.3. Radio Frequency Identification Applications (RFID) Data Collection

In this stage, we used RFID tags to collect the nurse movement data. The implementation of RFID applications can reduce the impact of disturbing nurses and protect patient privacy. There are four basic assumptions: (1) nurses are segmented and assigned into different sections in terms of duties; (2) the estimation of nurse traveling distance is obtained from the centroid of the reference tags at the location to the centroid of tags at another location; (3) the travel avoids obstacles, and; (4) the average speed is 4.5 km per hour.

**Figure 3:**
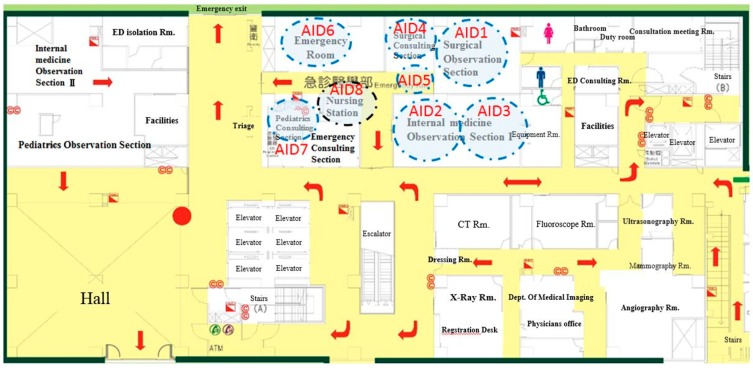
Radio frequency identification applications installed locations.

Eight Activator Identification (AID) zones denoted the most frequently traveled areas by nurses (Figure 3). AID1 was installed in the surgical observation section. Due to the limitation of the receiving range, two AID zones (AID2 and AID3) were in the first observation section. AID4 was set in the surgical consulting section; AID5 was closed to AID1 and AID4; AID6 was installed in the emergency room; AID7 was in the pediatrics consulting section; AID8 was in the nursing station and sent the information back to the server. For reducing noises or weak signals, the installed locations were tested.

The movement of nurse travel paths through ED facilities was collected by RFID tags, and we found the frequent travel paths by the Pareto Chart. It showed that the top five paths accounted for 82.88% of the total travel path. Therefore, we would target these five paths for the purpose of improvement. The paths of travel frequency in descending order were AID2–AID3, AID4–AID5, AID2–AID8, AID6–AID8, and AID7–AID8.

### 3.4. Facility Layout Improvement Process

In this stage, we first discovered the relationship between each facility unit based on the closeness rating. We classified five closeness ratings to measure the importance of relationships in our study, which were absolutely important (A), extremely important (I), important (I), unimportant (U), and undesirable (X). In the panel meeting, we discussed with the ED staff the segmenting of the ED facility into 13 sections. In the discussion, nine reasons with reason codes for closeness ratings were identified:

1. Use a common record
2. Share the same medical staff
3. Share the same space (observation section, warehouse, emergency room, etc.)
4. Medical staff contacts two departments at the same time
5. On paper or computer work contact
6. Patient visit process in the order of the two departments
7. Perform similar medical work
8. Use the same medical device
9. Unpleasant taste, dryness, or infection, etc.

**Figure 4:**
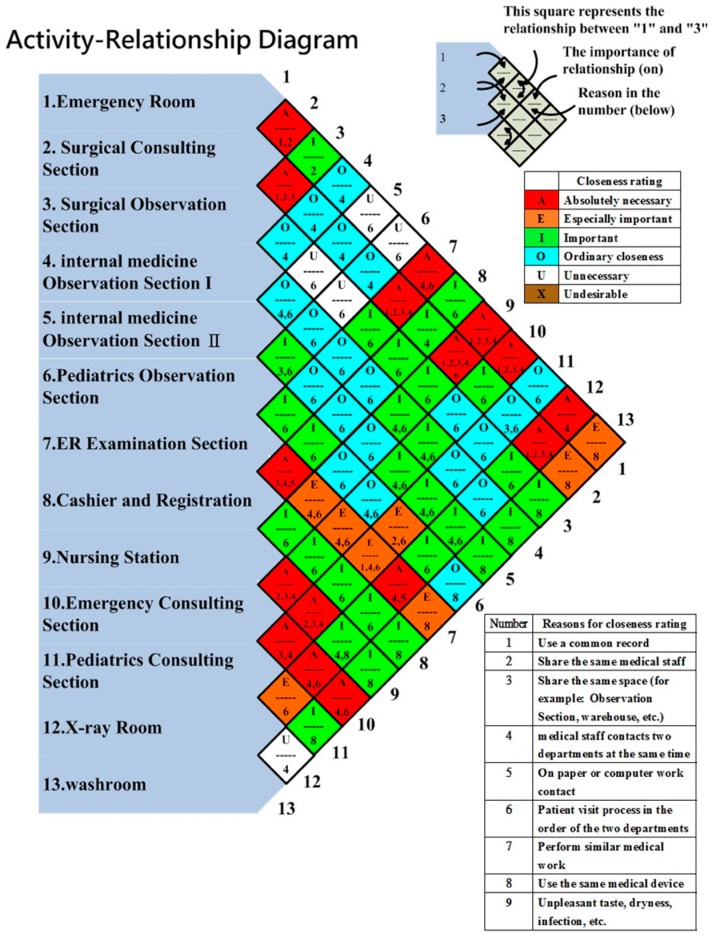
Activity-Relationship diagram.

The activity-relationship diagram, based on 13 sections, six closeness ratings, and nine reasons for closeness rating, is drawn in Figure 4. The objective of using an activity-relationship diagram was to find better layouts. The original design can be found in [42]. The first column indicates all of the sections in ED. The second column represents the relationship between the emergency room and the surgical consulting section, the surgical consulting section and the surgical observation section, the surgical observation section and the internal medicine observation section I, the internal medicine observation section I and the internal medicine observation section II, etc. The third column indicates the relationship between the emergency room and the surgical observation section, the surgical consulting section and the internal medicine observation section I, the surgical observation section and the internal medicine observation section II, etc. Each rhombus is divided into two halves; the upper half contains the rating letter, while the lower half contains the reason code. For example, the top rhombus of the second column showed that the medical staff used a common record and both worked in the emergency room and surgical consulting section, and the importance of the relationship was “Absolutely necessary”. Therefore, these two sections should be next to each other. The green square next to the top one, which was located in the third column, indicated that the emergency room and the surgical observation section shared the same medical staff, and the importance of the relationship was “Important”. In the diagram, 16 squares were identified with the highest closeness rating, while five squares were listed as unnecessary. Based on the activity-relationship diagram, we designed four ED dimensionless block diagrams.

## 4. Results

### 4.1. Model Validation

After we developed the model, *t*-test was used to validate it. We hypothesized that the critical indicators in the simulation model, which were ED patient’s length of stay (LOS) greater than 24, 48, or 72 h, did not have significant differences with the historical information. The average rate of LOS greater than 24 h in the model was 1.7%, where the actuality was around 1.58%; The average rate of LOS greater than 48 h in the model was 0.48%, where the actuality was around 0.47%; The average rate of LOS greater than 72 h in the model was 0.19%, where the actuality was around 0.18%. The results indicated that there was no statistically significant difference at the alpha level of 0.05. Therefore, the model was valid. The results are shown in Table 3.

### 4.2. Facility Design Strategies

**Figure 5:**
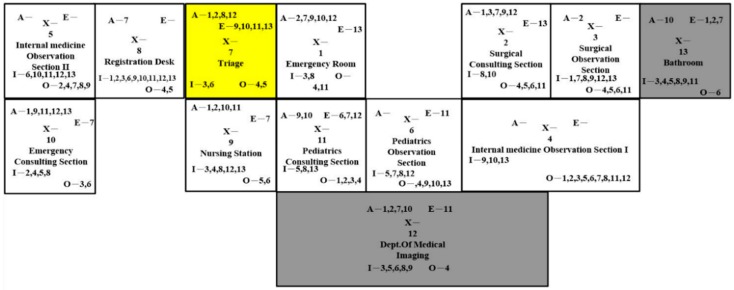
Layout Design A.

There were four design strategies regarding the activity-relationship diagram. In Figure 5, we made two changes in the first layout compared to the current layout, which involved (1) switching the location of the emergency consulting section and pediatrics observation section and (2) switching the location of the nursing station and pediatrics consulting section. There were two advantages of this design. The first advantage was that the pediatrics consulting section and pediatrics observation section were close to each other so the nurses could attend to the pediatric patients altogether. The second advantage of changing the layout was that the internal medicine patients would have a shorter distance to travel to the second internal medicine observation section. There were also shortcomings. The nurses would travel longer from the nursing station to the emergency consulting section, the first internal medicine observation section, and the surgical consulting section.

**Figure 6:**
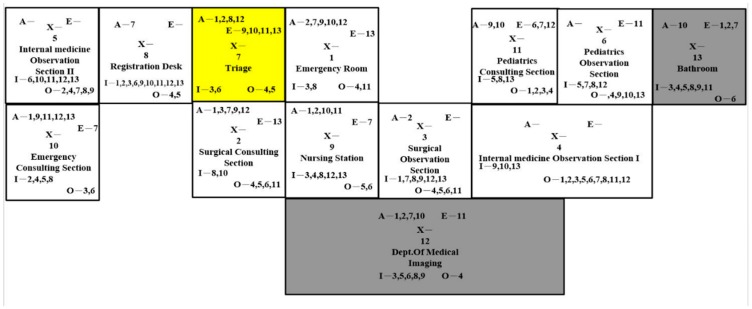
Layout Design B.

The design in Figure 6 is similar to the layout in Figure 8. However, we switched the location of the surgical section with the pediatrics section. In addition, we kept the nursing station at the same location as the original layout. Nurses could attend to the patients more conveniently from the merge of the periodic section and the merge of the surgical section. Nevertheless, they would travel longer from the nursing station to the emergency consulting section. The new location of the surgical observation section might have affected walkways.

**Figure 7:**
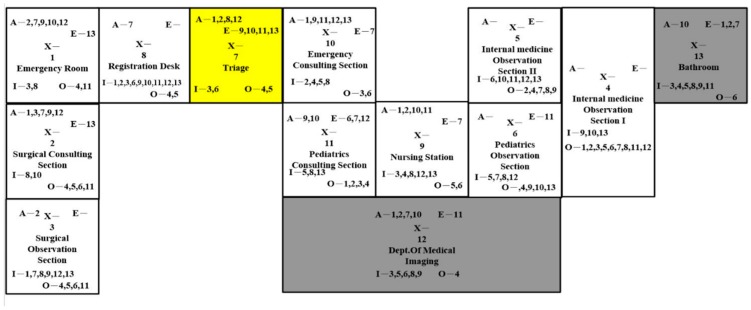
Layout Design C.

In Figure 7 the emergency room and the surgical section were close to each other on the left side. The first and second observation sections were merged, and the periodic consulting section and periodic observation section were close to the nursing station bilaterally. Nurses could gain benefits from the merging of the same division. The same traveling problems also appeared in this layout design.

**Figure 8:**
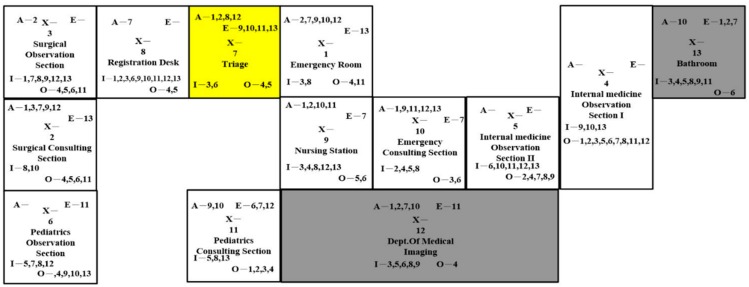
Layout Design D.

The last layout design involved merging the surgical sections and switching it with the second observation section (Figure 8). The changes were for nurses to attend patients easier. Moreover, the emergency room was expanded for more occupancy. This might have affected the space of the surgical observation section.

### 4.3. Experimental Results

We inputted these four designs of dimensionless block diagrams (Design A, Design B, Design C, and Design D) and calculated the patient travel paths into our simulation model. We assumed that the average patient travel speed was 4.5 km per hour. We expected that the new designs would not affect the patient flows in a negative way. The results are shown in Table 4. We found that the average patient travel distance and time increased for all of the designs. The results indicated that Design D was the only layout with less than 10% of increment in time and distance. Although the results indicated that the patients would not gain benefits from the new designs, the changes were relatively small. Patients would not be affected in the facility redesign.

We also calculated the total nurse travel distance by multiplying the frequency of travel paths. We assumed that the average nurse travel speed was 4.5 km per hour. The results are shown in Table 5. The first three designs did not have a positive improvement in average travel distance/time, and these designs also increased the patient travel time/distance. However, we found that Design D improved around 4% in travel distance, which meant decreasing 2.2 km per day. It was shown that switching the location of the second observation section with the surgical observation section and surgical consulting section would improve the efficiency of nurse work. In other words, reducing the travel distance/travel time could enhance the utilization of human resources, which represented higher efficiency in this study.

## 5. Conclusions and Discussions

Although a nurse has been assigned job duties in the fixed section, she often needs to support the work of others surrounding her, especially in ED. Improving efficiency cannot be solved simply by reassigning jobs. It is more critical to reduce waste from unnecessary traveling. Thus, the ED facility design tremendously affects the daily travel distance.

The majority of previous ED facility design research has focused on how to improve patient flows. In our study, our contribution was to reduce the traveling distance of nurses according to the association of facility sections. Moreover, we overcame the difficulty of collecting nurse data by adopting RFID applications. Not only did we eliminate human errors in data collection and inputs, but the implementation is also able to assist the decision-maker in managing and improving the flow more effectively. In addition, the new classification of ED facility sections can gather digital data in a convenient way. We used an activity-relationship diagram and simulation model to compare several scenarios. In the results, nurse utilization improved by 4% in the best scenario, which was 2.2 km of traveling distance or 140 min of traveling time reduction.

We suggested that the work schedule of other medical staff should be considered in future studies. Furthermore, the nursing station is the section with the most frequent traveling. It is also an interesting topic to distribute the nursing station.

In conclusion, the current ED facility cannot handle the increasing number of patients. Therefore, the emergency design guide has to be updated to meet the changes and needs flexibility and efficiency. The redesign of the ED facility would improve the health care quality of patients and the efficiency of treatment flows so that the ED crowding problem can be solved.

## Figures and Tables

**Table 1:** Simulation model inputs with real data.

Model Inputs	Value in Minutes [Lower 95% CL, Mean, Upper 95% CL]
Triage processing time	(5.23, 5.27, 5.31)
Triage (first aid patient) processing time	(30.55, 30.86, 31.16)
First aid processing time	(10.23, 10.59, 10.96)
Treatment processing time	(7.02, 7.26, 7.50)
Observation time	(389.34, 398.03, 406.71)
Observation time(Pediatric)	(255.72, 269.55, 283.39)

**Table 2:** Simulation model inputs with assumptions.

Model Inputs	Distributional Assumptions in Minutes *
Blood test processing time	Triangular (13, 14, 15)
X-ray processing time	Triangular (5, 6, 7)
MRI processing time	Triangular (30, 32, 35)
Other tests processing time	Triangular (10, 15, 20)

* The setting of distributional assumptions are suggested by the nurses.

**Table 3:** Validation Results.

Critical Indicators	μ of Actuality ^1^	μ of Simulation Model ^2^	σ of Simulation Model ^3^	*t*-Value	*p*-Value
ED patient’s length of stay greater than 24 h	1.58%	1.70%	1.64%	1.4524	0.1464
ED patient’s length of stay greater than 48 h	0.47%	0.48%	0.48%	0.3253	0.7450
ED patient’s length of stay greater than 72 h	0.18%	0.19%	0.18%	0.1718	0.8636

^1^ μ of Actuality indicates the average rate of the indicator gathered from the historical data. ^2^ μ of Simulation model indicates the average rate of the indicator obtained from the simulation model. ^3^ σ of Simulation model represents the standard deviation of the indicator in the simulation model.

**Table 4:** Simulation results of the patients.

Item	Average Travel Distance of Patient (meter)	Average Travel Time of Patient (min)	Improvement
Base	69.72	0.93	
Design A	90.02	1.20	−29%
Design B	89.63	1.20	−29%
Design C	83.17	1.11	−19%
Design D	73.62	0.98	−6%

**Table 5:** Simulation results of the nurses.

Item	Average Travel Distance of Nurse (meter)	Average Travel Time of Nurse (min)	Improvement
Base	2295.40	30.61	
Design A	2674.31	35.66	−17%
Design B	2391.26	31.88	−4%
Design C	2692.08	35.89	−17%
Design D	2206.43	29.42	4%
